# PCANet-Based Structural Representation for Nonrigid Multimodal Medical Image Registration

**DOI:** 10.3390/s18051477

**Published:** 2018-05-08

**Authors:** Xingxing Zhu, Mingyue Ding, Tao Huang, Xiaomeng Jin, Xuming Zhang

**Affiliations:** 1 Department of Biomedical Engineering, School of Life Science and Technology, Ministry of Education Key Laboratory of Molecular Biophysics, Huazhong University of Science and Technology, No. 1037, Luoyu Road, Wuhan 430074, China; D201677473@hust.edu.cn (X.Z.); myding@hust.edu.cn (M.D.); xmjin@hust.edu.cn (X.J.); 2 State Food and Drug Administration Hubei Center for Medical Devices Quality Supervision and Testing, No. 507, Hi-Tech Avenue, Donghu Hi-Tech Development District, Wuhan 430075, China; htao2010@126.com

**Keywords:** medical image registration, PCANet, structural representation, similarity metric, target registration error

## Abstract

Nonrigid multimodal image registration remains a challenging task in medical image processing and analysis. The structural representation (SR)-based registration methods have attracted much attention recently. However, the existing SR methods cannot provide satisfactory registration accuracy due to the utilization of hand-designed features for structural representation. To address this problem, the structural representation method based on the improved version of the simple deep learning network named PCANet is proposed for medical image registration. In the proposed method, PCANet is firstly trained on numerous medical images to learn convolution kernels for this network. Then, a pair of input medical images to be registered is processed by the learned PCANet. The features extracted by various layers in the PCANet are fused to produce multilevel features. The structural representation images are constructed for two input images based on nonlinear transformation of these multilevel features. The Euclidean distance between structural representation images is calculated and used as the similarity metrics. The objective function defined by the similarity metrics is optimized by L-BFGS method to obtain parameters of the free-form deformation (FFD) model. Extensive experiments on simulated and real multimodal image datasets show that compared with the state-of-the-art registration methods, such as modality-independent neighborhood descriptor (MIND), normalized mutual information (NMI), Weber local descriptor (WLD), and the sum of squared differences on entropy images (ESSD), the proposed method provides better registration performance in terms of target registration error (TRE) and subjective human vision.

## 1. Introduction

Nonrigid multimodal image registration is very important for medical image processing and analysis. Due to the different principles of various imaging technologies, such as ultrasound (US), computed tomography (CT), magnetic resonance (MR) imaging, and positron emission tomography (PET), they have their own advantages in reflecting anatomical or functional information of the human body. Multimodal image fusion can combine information of different modalities to facilitate disease diagnosis and treatment. Image registration, which aims to find the correct spatial alignment between the corresponding structures in images, is a prerequisite process for effective image fusion and has been widely studied. For example, the registration of the transrectal ultrasound image (TRUS) with the preoperative MR image has been widely studied for the guidance of prostate biopsies [1,2]. In craniomaxillofacial surgery [3], it is necessary to align the intraoperative images with the three-dimensional (3D) virtual model in the surgical navigation. In the treatment of epilepsy [4], the registration of MRI and functional imaging modalities, such as PET, is implemented to assist in identifying the functionally eloquent regions of brain tissue and guide the placement of the electrode.

For image registration, the similarity metrics play an important role in ensuring accurate registered results. However, it is a challenge to find an effective similarity metric for multimodal image registration. There are many widely used similarity metrics, such as sum of squared differences (SSD) and cross-correlation [5], but such metrics are not suitable for multimodal medical image registration directly due to the different intensities of the multimodal images. Mutual information (MI) [6], which aims to find the statistical intensity relationship across images, has been widely used in multimodal image registration. However, this method is very time-consuming, and it is likely to produce misalignment because MI is not a convex function and it is easy to get into the local optimum [7]. Various improved MI metrics have been presented to address these problems, including normalized mutual information (NMI) [8,9], regional mutual information [10,11], conditional mutual information [12], and self-similarity weighted mutual information [13]. However, these methods ignore the local structural information, which may lead to the degraded registration performance especially in the edge regions.

To overcome the drawback of the above-mentioned similarity metrics, structural representation (SR) methods have been investigated by firstly transforming a multimodal registration into a mono-modal one using the SR method and then computing the SSD between the SR results. The local gradient orientation is utilized to find correspondences across image modalities [14,15]. However, the gradient estimation is sensitive to noise. The Weber local descriptor (WLD)-based SR method has been presented by Yang et al. [16]. However, this method is sensitive to image noise and sometimes it cannot provide consistent structural representation results for the same organs in multimodal images. Heinrich et al. have proposed the modality-independent neighborhood descriptor (MIND) [17] based on the principle of local self-similarity utilized in nonlocal means denoising. Although this descriptor is robust to noise, intensity differences, and non-uniform bias fields, it is not provided with rotational invariance. Wachinger et al. [18] have proposed the construction of structural representations based on entropy images. This method estimates the probability density function of image patches centered on the pixel and then constructs entropy images based on Shannon’s theorem. However, the entropy images tend to be fuzzy, which may result in the inaccurate computation of similarity metrics. The idea of dimensionality reduction has been also used for image registration [19] and the construction of structural representation based on dimensionality reduction has been studied extensively. Structural representation based on Laplacian eigenmaps is proposed in [18]. In this method, a neighborhood graph is constructed based on all image patches, and it is utilized to calculate the Laplacian graph to determine the low-dimensional embedding. The structural representation is produced by aligning the embeddings of different modalities. Compared with an entropy image, Laplacian image looks more like the original image and thus its appearance image across the modalities is more consistent. The disadvantage of Laplacian image lies in its high computational cost and vulnerability to image noise. Piella has proposed to use the image intensities and position of pixels to construct diffusion maps for multimodal image registration [20]. The registration performance of this method is influenced by the adoption of the first diffusion coordinate, which only represents the coarse geometry of the image. A probabilistic edge map (PEM) that is generated from a structured decision forest has been proposed by Oktay et al. for multimodal ultrasound image registration [21]. In general, these SR methods are mostly based on human-designed low-level features, which do not properly represent the complicated characteristics of the variety of medical images.

Learning the features automatically from data of interest provides a plausible solution to accurate structural image representation. Deep learning, as a popular machine learning method, is well-suited for automatic feature learning. The various deep learning models, such as deep belief network (DBN), convolutional neural network (CNN), and stacked autoencoder (SAE), can extract intrinsic features from a large number of data using multilayer linear and nonlinear transformation. Recently, deep learning has been utilized for image registration using two kinds of methods. The first kind of method extracts the image features using such networks as CNN and SAE and uses them in traditional registration methods to produce the registered images [22,23,24]. In these methods, the improper selection of parameters in CNN and SAE may lead to locally optimal registered results. Furthermore, these deep learning models generally involve a slow learning rate. The second kind of method uses deep learning to realize end-to-end image registration by learning the deformation field directly from the input images. Deformable image registration methods based on CNN are proposed in [25,26]. However, these methods are not suitable for multimodal image registration. Hu et al. [27] and Hessam et al. [28] extended CNN to multimodal deformable image registration. Due to the adoption of supervised learning in these methods, their performance was influenced by the scarcity of the labeled medical imaging data and the inaccurate labeled training samples produced by the traditional registration method for training the CNN.

To address these problems, this paper has proposed to realize the structural representation for image registration based on PCANet. PCANet, proposed by Chan et al. [29], involves an input layer, hidden layers, and an output layer. This network utilizes a principal component analysis (PCA)-based unsupervised learning method working on image patches to produce the network parameters in hidden layers. Compared with CNN, PCANet can be trained much more easily and it requires no labeled data for network training. However, this network uses binary hashing in the output layer to produce the final output, which may lead to the loss of some image features in the output. Therefore, the improved version of PCANet is proposed in this paper to realize structural image representation. In the proposed registration framework, the improved PCANet-based structural representation (PSR) method is used to extract the multilevel features of medical images and the extracted features are combined to produce the structural representation results. The L2 distance between the PSR results of images is used as the similarity metric. By using the free-form deformation (FFD) [30] model as the transformation model, the Limited memory Broyden–Fletcher–Goldfarb–Shannon (L-BFGS) [31] is used to optimize the similarity metrics to obtain the parameters of the transformation model. Extensive experiments have been conducted on simulated and real medical images to appreciate the registration performance of the proposed method in terms of the target registration error (TRE).

This paper is organized as follows: Section 2 describes the construction of PSR and the implementation of the proposed nonrigid multimodal registration method. Section 3 presents the parameter settings in the proposed method and a comparison of its registration performance with that of the state-of-the-art registration methods. Finally, conclusions and future research directions are given in Section 4.

## 2. Methods

### 2.1. Structure of the Improved PCANet

**Figure 1:**
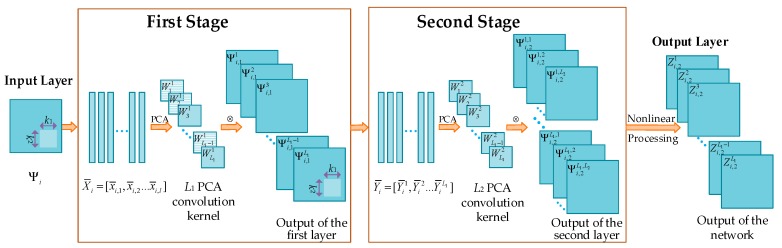
Diagram of two-layer PCANet.

For the traditional PCANet, binary hashing is used in the output layer, where the output values are set to be 1 and 0 for positive entries and other entries, respectively. However, the negative entries also carry the structural information of the image. Therefore, the loss of some image features may be involved in the output of the traditional PCANet. The improved PCANet network addresses this problem by using the absolute values of the outputs at the second layer and the sigmoid function for nonlinear processing. The improved PCANet model with two hidden layers is illustrated in Figure 1. In what follows, we will describe the structure of the improved PCANet in detail.

#### 2.1.1. The First Stage of PCANet

For each pixel in the *i*-th image of size m×n in *N* training images ${\left\{\Psi_{i}\right\}}_{i=1}^{N}$, a patch of size $k_{1}\times k_{2}$ is extracted and all image patches are collected. Accordingly, the number of patches for the image $\Psi_{i}$ will be $l=(m-[k_{1}/2])\times (n-[k_{2}/2])$, where [k] represents the smallest positive integer greater than or equal to k. These patches are represented with $x_{i,1},x_{i,2},\ldots x_{i,l}\in R^{k_{1}\times k_{2}}$, where $x_{i,j}$ denotes the *j*-th patch in $\Psi_{i}$. The patch mean is subtracted from each image patch and the mean-removed patch is vectorized. The resultant set of vectors is arranged into a matrix ${\bar{X}}_{i}=[{\bar{x}}_{i,1},{\bar{x}}_{i,2},\ldots {\bar{x}}_{i,l}]$. For all training images, the matrices are constructed in the same way and put together to yield:

(1) $$\bar{X}=[{\bar{X}}_{1},{\bar{X}}_{2},\ldots {\bar{X}}_{N}]\in R^{k_{1}k_{2}\times Nl}.$$

PCA is utilized to remove the redundant information of $\bar{X}$ by finding an orthonormal subspace whose bases are along the directions of maximum variance in the data. The solution is known as the principal eigenvectors of $\bar{X}{\bar{X}}^{T}$. The eigenvalues of $\bar{X}{\bar{X}}^{T}$ are sorted in decreasing order:

$$\lambda_{1}\geq \lambda_{2}\geq \lambda_{3}\ldots \geq \lambda_{d}$$

where d denotes the number of the eigenvalues of $\bar{X}{\bar{X}}^{T}$.

Let the number of selected filters at the first layer be $L_{1}$. The first $L_{1}$ eigenvectors are chosen and mapped into $L_{1}$ matrices to produce the convolution kernel $W_{j}^{1}$ as:

(2) $$W_{j}^{1}=map(p_{j}(\bar{X}{\bar{X}}^{T}))\;(j\in [1,L_{1}])$$

where $p_{j}(\bar{X}{\bar{X}}^{T})$ denotes the eigenvector corresponding to the *j*-th eigenvalue. The map is a function for rearranging the vector of $k_{1}\times k_{2}$ elements into a matrix, where the first $k_{1}$ elements are arranged in the first column, and the $k_{1}+1$ to $2k_{1}$ elements are arranged in the second column, and so on. The j-th filter’s output for the i-th training image at the first stage is:

(3) $$\Psi_{i,1}^{j}=\Psi_{i}⊗W_{j}^{1}$$

where ⊗ denotes two-dimensional (2D) convolution. To ensure that $\Psi_{i,1}^{j}$ has the same size as $\Psi_{i}$, $\Psi_{i}$ is zero-padded before convoluting it with $W_{j}^{1}$.

At the first stage, PCANet will produce $L_{1}$ outputs for each input image and they will be used as the input of the second stage.

#### 2.1.2. The Second Stage of PCANet

To capture higher-level features, the multiple stages of PCA will be stacked as similar to DBN. Similar to the first stage, a $k_{1}\times k_{2}$ image patch centered at each pixel in $\Psi_{i,1}^{j}$ is considered. All image patches from the outputs ${\left\{\Psi_{i,1}^{j}\right\}}_{i=1}^{N}$ are then mean-removed, vectorized, and concatenated to produce a matrix denoted as ${\bar{Y}}^{j}=[{\bar{Y}}_{1}^{j},\;{\bar{Y}}_{2}^{j},\;{\bar{Y}}_{3}^{j}\ldots \;{\bar{Y}}_{N}^{j}]\;\in R^{k_{1}k_{2}\times Nl}$. All the matrices ${\bar{Y}}^{j}$ are concatenated to generate the matrix $\bar{Y}=[{\bar{Y}}^{1},{\bar{Y}}^{2},{\bar{Y}}^{3},\ldots {\bar{Y}}^{L_{1}}]$. The eigenvector of $\bar{Y}{\bar{Y}}^{T}$ is calculated and the eigenvalues are sorted in decreasing order. The $L_{2}$ eigenvectors are selected and mapped into the convolution kernel $W_{k}^{2}$ of the second stage in PCANet:

(4) $$W_{k}^{2}=map(p_{k}(\bar{Y}{\bar{Y}}^{T}))\;(k\in [1,L_{2}]).$$

The output of this stage is:

(5) $$\Psi_{i,2}^{j,k}=\Psi_{i,1}^{j}⊗W_{k}^{2}\;(k\in [1,L_{2}]).$$

For the second stage, each input image will produce $L_{2}$ outputs. This process can be repeated to produce the deeper architecture, thereby providing better feature representation results.

#### 2.1.3. Output Stage

All of the outputs at the second stage will be transformed using the sigmoid function as:

(6) $$Z_{i,2}^{j}=\sum_{k=1}^{L_{2}}2^{\left(8-k\right)}\{S(|\Psi_{i,2}^{j,k}|)-S(0)\}\;(j\in [1,L_{1}])$$

where $S(x)=\frac{1}{1+e^{-\alpha x}}$ with α denoting a constant predefined as 0.005. The item S(0) is subtracted to make sure that the background of the image is zero. The reason for using the absolute value of $\Psi_{i,2}^{j,k}$ in Equation (6) is that the negative values in the outputs also carry the structural information of the image and accurate structural image representation cannot be achieved if these values are abandoned. In Equation (6), the weight $2^{\left(8-k\right)}$ is utilized for two reasons. The first reason is that the intensities of the input image are between 0 and 255 and the gray level of the features should be consistent with that of the input image. The second one is that, according to the theory of PCA, the larger the eigenvalue is, the more information the corresponding eigenvector carries, and thus the weight should be greater.

### 2.2. Structural Representation

**Figure 2:**
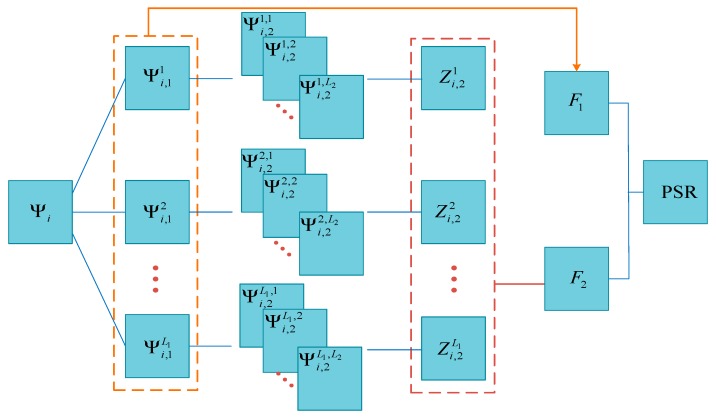
Scheme for constructing a PCANet-based structural representation (PSR) by using the multilevel features produced by PCANet.

Inspired by the previous work in [32], the multilevel information extracted by PCANet is processed separately and exploited to construct the structural image representations. The structural representation of input image $\Psi_{i}$ is computed based on the outputs produced at the first stage and the network outputs in the improved PCANet. Figure 2 shows the construction of PSR using the multilevel features produced by PCANet. For the first stage, the sum of squared pixel intensities in all output images is calculated and a fused feature image is produced based on the calculated result and the number of filters:

(7) $$F_{1}=\frac{1}{L_{1}^{2}}\sum_{j=1}^{L_{1}}{\left(\Psi_{i,1}^{j}\right)}^{2}$$

For the output stage, the fused feature image is generated in the same way as in the first stage:

(8) $$F_{2}=\frac{1}{L_{1}^{2}}\sum_{j=1}^{L_{1}}{\left(Z_{i,1}^{j}\right)}^{2}$$

Finally, the multilevel features are combined using the exponential function to construct the PSR as:

(9) $$\mathrm{PSR}=\exp(-\frac{F_{1}}{h_{1}})\exp(-\frac{F_{2}}{h_{2}})$$

where the decay parameters $h_{1}$ and $h_{2}$ should ensure that there will be a high response in PSR for similar regions in the images to be registered and a low response for dissimilar regions.

The decay parameters are locally adaptive for each image pixel at (u,v). The pixel-wise parameters $h_{s}(u,v)\left(s=1,2\right)$ are defined as:

(10) $$h_{s}(u,v)=[c_{1}\sigma_{s,1}(u,v)+c_{2}\sigma_{s,2}){]}^{2}$$

where $\sigma_{s,1}(u,v)$ is the local variation in the image, $\sigma_{s,2}$ is the global threshold, and $c_{1}$ and $c_{2}$ are constants which are used to control the decay parameters. The local variation has two effects. One is that the different pixels should have a different response to the extracted features, and the other is to ensure that all image pixels have the same order of response magnitudes. If there is no global threshold, a small value of local variation will yield a sharp decay function in the smooth regions.

Generally, in nonlocal means image denoising, the decay parameter is proportional to the noise variance [33]. Borrowing from this idea, $h_{1}$ is chosen to be related to the gradient of the image. For $h_{1}$, an eight-neighborhood *B* of a pixel at (u,v) is selected. The parameters $\sigma_{1,1}(u,v)$ and $\sigma_{1,2}$ are computed as:

(11) $$\sigma_{1,1}(u,v)=\frac{\left|8\Psi_{i}(u,v)-\sum_{(a,b)\in B}\Psi_{i}(a,b)\right|}{8}$$

(12) $$\sigma_{1,2}=mean(\sigma_{1,1}),\;s.t.\;\sigma_{1,1}\neq 0$$

where mean(·) denotes the mean operator. Because the outputs at the second stage are calculated from those at the first stage, $h_{2}$ should be related to the gradient of the output images at the first stage. However, many images are produced at the first stage, which makes it difficult to determine $h_{2}$. A feasible way for determining $h_{2}$ is to use the mean of all output images in the second layer, i.e.,

(13) $$\sigma_{2,1}(u,v)=\frac{\left|\sum_{j=1}^{L_{1}}Z_{i,2}^{j}(u,v)\right|}{L_{1}}$$

(14) $$\sigma_{2,2}=mean(\sigma_{2,1}),\;s.t.\;\sigma_{2,1}\neq 0\;.$$

### 2.3. Similarity Metric and Cost Function

PSR can be used to convert the multimodal image registration into a mono-modal one, which means that the similarity of multimodal images can be evaluated by a simple similarity metric. In this paper, the similarity metric $D(I_{r},I_{f})$ between the reference image $I_{r}$ and the floating image $I_{f}$ is defined as the SSD between their PSR results.

(15) $$D(I_{r},I_{f})=\frac{1}{mn}{\left\|PSR_{I_{r}}-PSR_{I_{f}}\right\|}_{2}^{2}$$

where ${\mathrm{PSR}}_{I_{r}}$ and ${\mathrm{PSR}}_{I_{f}}$ denote the PSR results of $I_{r}$ and $I_{f}$, respectively.

The FFD model is used as the transformation model. In order to find the optimal spatial transformation, a cost function which is associated with the spatial transformation parameters is defined. The cost function consists of the similarity metric and the smoothness constraint, and it is expressed as:

(16) $$C(I_{r},T(I_{f}))=D(I_{r},T(I_{f}))+\lambda C_{smooth}(T)$$

where λ is the penalty parameter which is used to balance the two components of the cost function. The smoothness constraint $C_{smooth}$ is used to constrain the spline-based FFD transformation and improve the robustness of the algorithm. For a two-dimensional image, $C_{smooth}$ is defined as:

(17) $$C_{smooth}=\frac{1}{mn}\int_{0}^{m}\int_{0}^{n}\left[{\left(\frac{\partial^{2}T}{\partial x^{2}}\right)}^{2}+2{\left(\frac{\partial^{2}T}{\partial x\partial y}\right)}^{2}+{\left(\frac{\partial^{2}T}{\partial y^{2}}\right)}^{2}\right]dxdy.$$

The cost function can be optimized using the Limited memory Broyden–Fletcher–Goldfarb–Shanno (L-BFGS) optimization algorithm to produce the optimal parameters of the FFD transformation T.

### 2.4. Implementation of the Proposed Registration Method

The implementation details of the proposed method can be summarized as the following five steps.

Step 1: Train the PCANet using a large amount of training data and obtain the convolution kernels of the two hidden layers;

Step 2: Calculate the structural representation result ${\mathrm{PSR}}_{I_{r}}$ of the reference image $I_{r}$ and ${\mathrm{PSR}}_{I_{f}}$ of the floating image $I_{f}$ according to Equations (7)–(9).

Step 3: Construct the cost function based on ${\mathrm{PSR}}_{I_{r}}$ and ${\mathrm{PSR}}_{I_{f}}$ as described in Equations (15)–(17).

Step 4: Minimize the cost function using the L-BFGS optimization algorithm to find the optimal spatial transformation.

Step 5: Output the optimal transformation T and get the registered image by transforming the floating image with the obtained T.

## 3. Experimental Results

In this section, several experiments have been performed on different datasets, including a simulated brain image dataset BrainWeb at http://brainweb.bic.mni.mcgill.ca/brainweb/, a real brain image dataset Atlas at http://www.med.harvard.edu/aanlib/home.html, and the CT and MR image dataset RIRE at http://www.insight-journal.org/midas/community/view/16. The parameter settings of the proposed method are firstly discussed based on the Atlas dataset. Then, the performance of the proposed method is compared with that of such state-of-the-art methods as MIND, ESSD, WLD, and NMI based on the BrainWeb and RIRE datasets.

**Figure 3:**
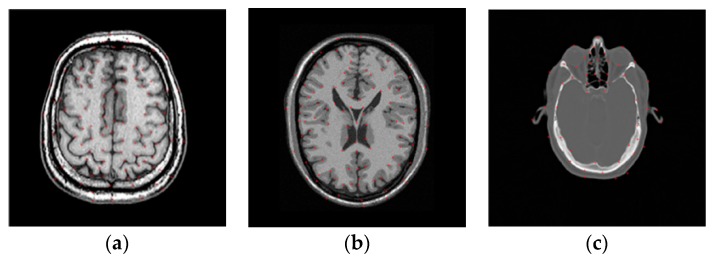
Distribution of landmarks in the reference images from the three datasets. (**a**) Atlas dataset; (**b**) BrainWeb dataset; (**c**) RIRE dataset.

For the appreciation of registration accuracy, the target registration error (TRE), which is the average Euclidean distance of these anatomical landmarks in the images to be registered, is used and it is defined as:

(18) $$TRE=\frac{1}{\left|M\right|}{\left\|r(M)-(f(M)+T_{R}(M))\right\|}_{2}$$

where M and |M| denote the set of anatomical landmarks and the number of landmarks selected in the reference image, respectively, and r(M) and f(M) represent the pixel position of the landmarks in the reference and floating images, respectively. The floating image is produced based on a B-spline function. Here, the B-spline control points are applied to the image and randomly displaced to generate a random deformation field, based on which the floating image is produced. $T_{R}$ is the estimated deformation obtained by the registration method. In this paper, the landmarks are selected manually based on the doctors’ advice. Figure 3 shows an example of the distribution of the chosen landmarks in MR images from the Atlas, BrainWeb, and RIRE datasets.

### 3.1. Parameter Settings

The main parameters of the PCANet include the number of stages, the number of filters in each stage, the patch size, and the decay parameters. In our experiments, it is found that two-layer PCANet is good enough for image registration, and a deeper network does not lead to much better performance. As regards the number of filters in each stage, it is set to $L_{1}=L_{2}=8$, which is inspired from the Gabor filters [34] with eight orientations. To determine the remaining two parameters, i.e., the patch size and the decay parameters, we will tune them based on the Atlas dataset. From this dataset, 100 images of size 256×256 are chosen. In order to ensure sufficient training data, we will perform four different nonrigid deformations on each image. The resultant 500 images are used to train the PCANet.

#### 3.1.1. Impact of the Patch Size

**Figure 4:**
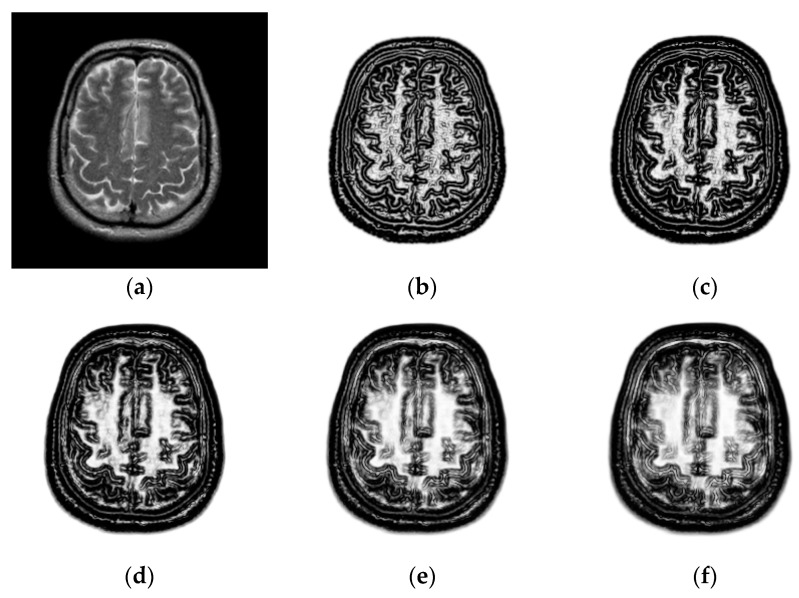
PSR with the different patch sizes. (**a**) T2-weighted magnetic resonance (MR) images in the Atlas dataset; (**b**) patch size: $k_{1}=k_{2}=3$; (**c**) patch size: $k_{1}=k_{2}=5$; (**d**) patch size: $k_{1}=k_{2}=7$; (**e**) patch size: $k_{1}=k_{2}=9$; (**f**) patch size: $k_{1}=k_{2}=11$.

**Figure 5:**
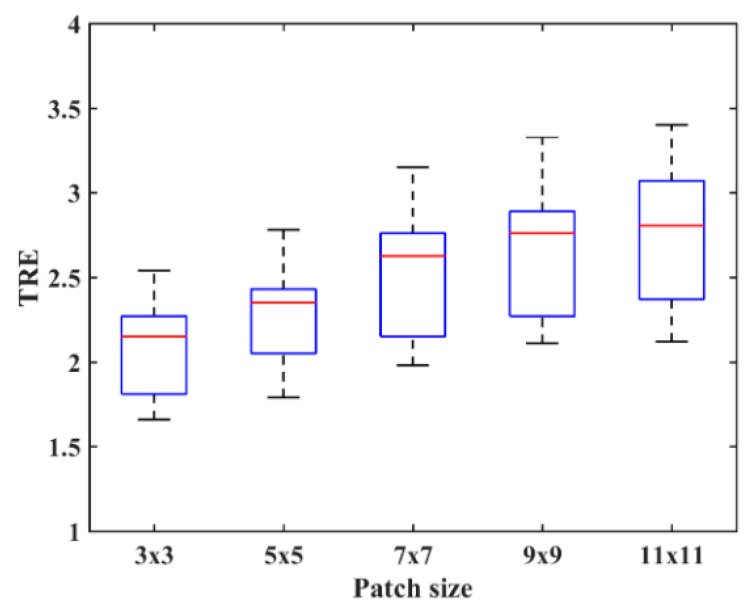
Target registration error (TRE) for the PSR method using the various patch sizes.

To explore the effect of patch size on image registration, $k_{1}$ and $k_{2}$ are changed from 3 to 11. Figure 4 shows the PSR of a T2-weighted image. We can see that the PSR becomes more blurry with the increasing of patch size. As shown in Figure 4d–f, the features will not be obvious and some weak features even disappear when the patch size is large, which is disadvantageous for image registration. To objectively evaluate the effect of patch size on image registration, the chosen 10 images will be aligned and their TRE values calculated. Figure 5 shows the TRE for our method using the various patch sizes. The observation from Figure 5 demonstrates that a too large patch size has a negative influence on registration accuracy, and the best registered result can be obtained when the patch size is 3×3.

#### 3.1.2. Impact of the Coefficients $c_{1}$ and $c_{2}$

**Figure 6:**
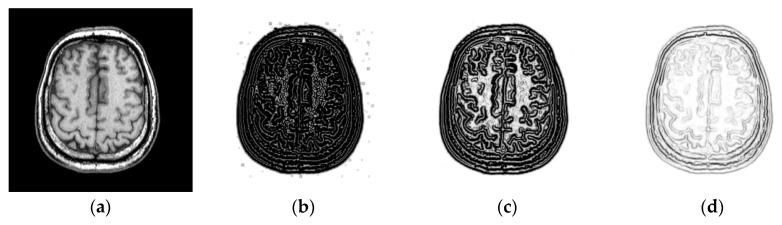
PSR of MR images by using the different thresholds. (**a**) T1-weighted MR image; (**b**) PSR with a small threshold; (**c**) PSR with an appropriate threshold; (**d**) PSR with a high threshold.

The coefficients $c_{1}$ and $c_{2}$ have an important influence on the PSR. When the two coefficients are small, this will lead to a sharp decay function and the values of PSR will be close to zero, which makes it difficult to distinguish the features in PSR. Higher values indicate a broader response, which is also disadvantageous for feature discrimination. Figure 6b–d show the PSR of a T1-weighted MR image using the different global thresholds. It can be seen from Figure 6b that a small global threshold will result in some false features and renders it difficult to distinguish the smooth region and the edge region. As shown in Figure 6d, some features are weakened and the less-obvious features in the image disappear due to the influence of a high threshold. By comparison, a suitable threshold will facilitate producing a clear structural representation as shown in Figure 6c.

**Figure 7:**
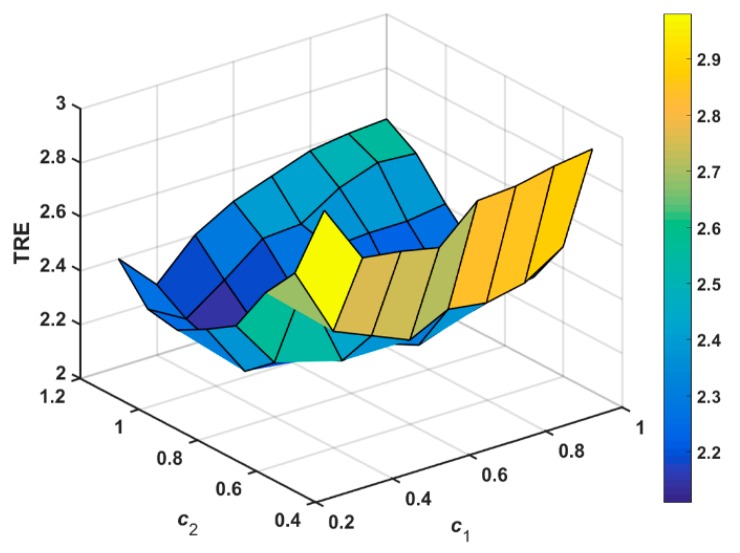
TRE for MR images using the different coefficients $c_{1}$ and $c_{2}$.

To evaluate the influence of the coefficients objectively, $c_{1}$ is varied from 0.2 to 1 and $c_{2}$ is varied from 0.4 to 1.2 with a step size of 0.1. The corresponding TRE results for the various coefficients are shown in Figure 7. It is easy to see from Figure 7 that relatively low TRE values can be obtained when $c_{1}$ is between 0.6 and 0.9 and $c_{2}$ is between 0.5 and 0.8. Based on the above analysis, we will set the coefficients $c_{1}$ and $c_{2}$ to be 0.8 and 0.6, respectively, to ensure good registration results.

### 3.2. Comparison with State-of-the-Art Registration Methods

**Figure 8:**
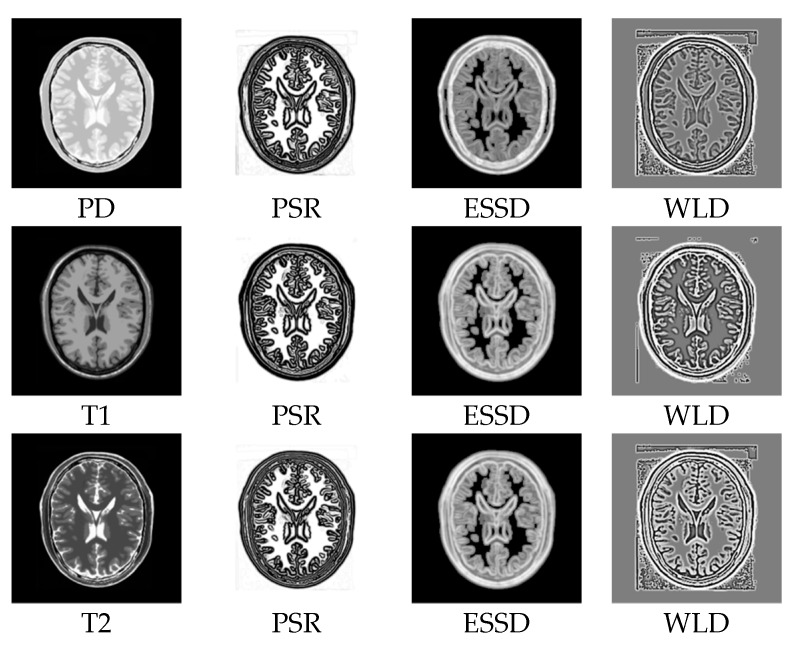
Structural representation results of different MR images. The first column corresponds to the PD-weighted, T1-weighed, and T2-weighed MR images, respectively. The second to fourth columns correspond to the representation results of MR images for PSR, ESSD, and WLD, respectively.

In order to demonstrate the superiority of our method, we compare PSR with the structural representations obtained by other methods on the BrainWeb dataset. Figure 8a–c show the PD-, T1-, and T2-weighted MR images. Figure 8d–f,g–i, and j–l show the structural representation results of these images for the proposed method, the ESSD method, and the WLD method, respectively. It can be seen from Figure 8 that the PSR result for our method is more consistent for multimodal images than the results from the other compared methods. Furthermore, a higher contrast exists between the smooth region and the edge region in the PSR. Compared with PSR, the result from the ESSD method seems to be blurry. As for the WLD method, it produces a lot of artifacts around the boundary and poorer consistency among the different modalities. For example, the intensities of the structural representation results of the same tissue in the T1 and T2 images differ greatly as shown in Figure 8k,l.

In addition to the comparison of the structural representations, a comparison of the registration accuracy between the proposed method and the state-of-the-art methods has also been made based on the BrainWeb dataset and the RIRE dataset. For the proposed method, we set the penalty parameter λ in the optimization algorithm to 0.01, the PCANet patch size to 3×3, the decay number parameters $c_{1}=0.8$ and $c_{2}=0.6$, and the number of filters in each layer $L_{1}=L_{2}=8$. For the compared methods, the related parameters are tuned to ensure the best registered results for the two datasets.

#### 3.2.1. Test on the BrainWeb Dataset

**Figure 9:**
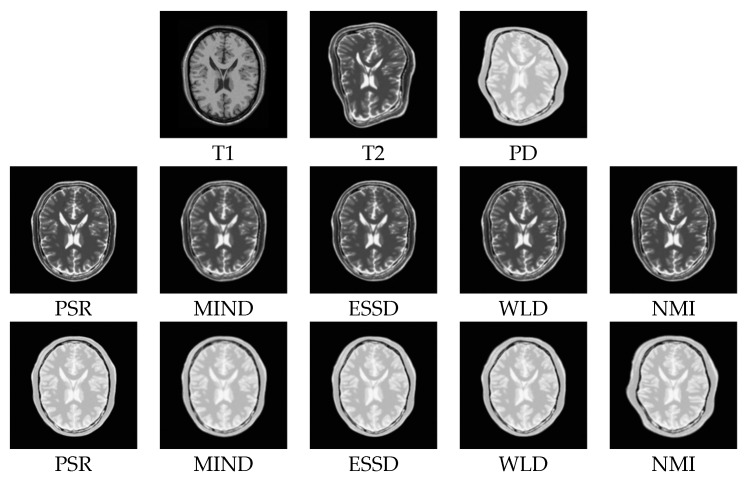
Registration results for the PSR, MIND, ESSD, WLD, and NMI methods performed on the BrainWeb dataset (T1–T2, T1–PD). The first row corresponds to the T1-weighted, T2-weighted, and PD-weighted MR images, respectively. The second row corresponds to the registered results of T1–T2 images for PSR, MIND, ESSD, WLD, and NMI, respectively. The third row corresponds to the registered results of T1-PD images for PSR, MIND, ESSD, WLD, and NMI, respectively.

**Figure 10:**
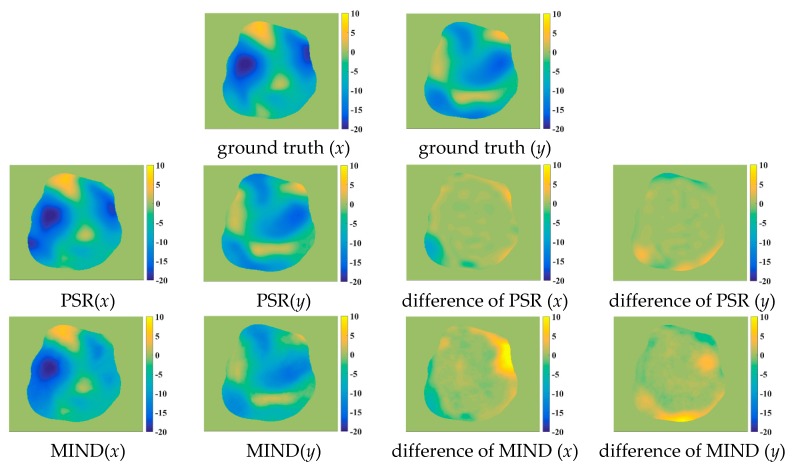
The deformation fields for the various methods. The first row is the ground truth of deformation in the *x* and *y* orientations, respectively; the second row is the deformation field of the PSR method and the difference between the PSR and ground truth in the *x* and *y* orientations, respectively; the third row is the deformation field of the MIND method and difference between the PSR and ground truth in the *x* and *y* orientations, respectively.

Figure 9 shows some registered results for the five compared methods. Obviously, these methods can correct the deformations to some extent. However, the NMI, WLD, and ESSD methods often fail to align the float and reference images with large deformations. By comparison, the registered results of the MIND and PSR methods are much closer to the reference image. Meanwhile, the proposed method has better registered results for the image details than the MIND method. For a more intuitive comparison, the deformation fields are shown in Figure 10. The ground truth of the deformation fields is the spatial difference between the reference and floating images. Clearly, the PSR method provides a closer deformation field to the ground truth than the MIND method.

In order to evaluate and compare the registered results objectively, 50 registration experiments have been conducted. Table 1 shows the mean and the standard deviation (std) of TRE values for all registration methods and ’/’ represents that no registration is implemented. The results indicate that the proposed method achieves higher registration accuracy than the other methods. Meanwhile, we can see that the proposed method is more stable than the other methods. A *t*-test is performed between the PSR method and each comparison method. The test results show that there exists a significant difference between the proposed registration method and any other compared method (*p* < 0.05). The reason for the superiority of the PSR method can be explained in this way. Medical images are complicated and it is difficult for human-designed descriptors to represent their complex features. However, due to the strong feature learning abilities of PCANet in the proposed method, more intrinsic features can be extracted automatically and effectively, thereby leading to better structural representation results.

**Figure 11:**
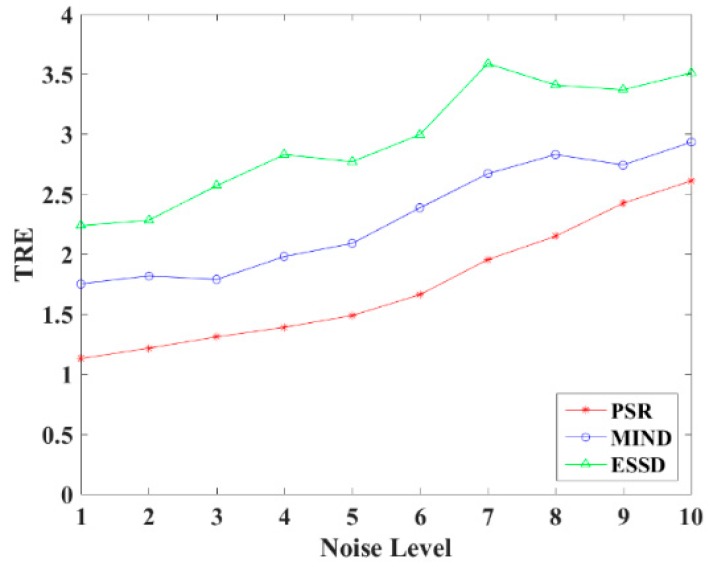
TRE for different noise levels.

**Figure 12:**
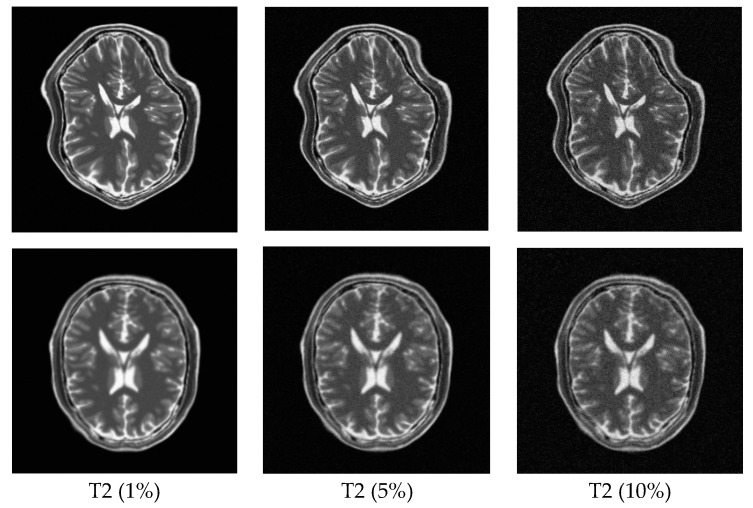
Registration results for T1–T2 images with different levels of Rician noise. The first row shows the T2-weighted floating images with 1%, 5%, and 10% Rician noise, respectively. The second row corresponds to the registered results for the PSR method.

In order to explore the robustness of the registration methods, different levels (1–10%) of Rician noise have been added to the MR images. Figure 11 shows the TRE of the PSR, MIND, and ESSD registration methods and Figure 12 shows some registered results of T1–T2 images with different levels of Rician noise for PSR. The result shows that with increasing levels of noise, the registered results will become worse to some extent, but as a whole, our method is robust to the noise.

#### 3.2.2. Test on the RIRE Dataset

**Figure 13:**
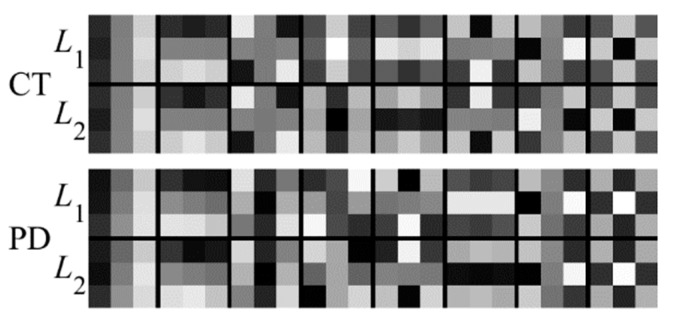
Filters learned for the multiple layers by the PCANet. The first and second rows are the filters at the first and second stages for the CT images, respectively; the third and fourth rows are the filters at the first and second stages for the PD images, respectively.

To further test the registration performance of the proposed method, experiments have been conducted on the RIRE dataset. Figure 13 shows the filters learned by the PCANet. It can be seen from Figure 13 that the PCANet can learn the different convolution kernels for the CT and MR images, which can facilitate effective feature extraction for the different images.

**Figure 14:**
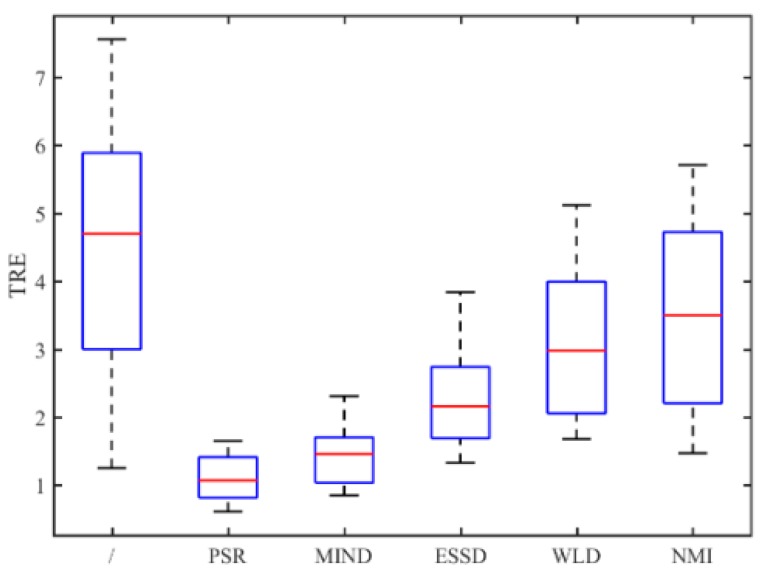
TRE for all registration methods performed on the RIRE dataset.

**Figure 15:**
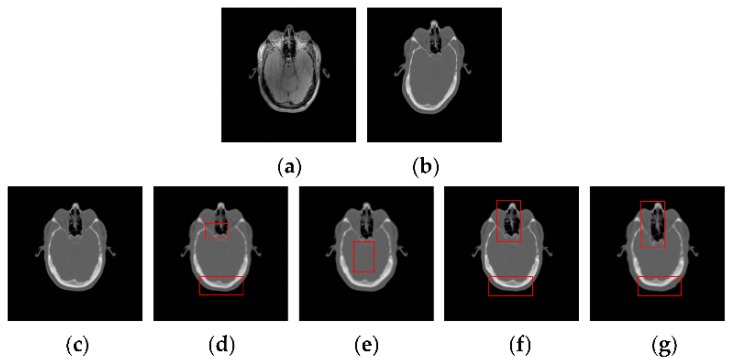
CT-MR image registration results for the PSR, MIND, ESSD, WLD, and NMI methods. (**a**) The reference PD image; (**b**) The floating CT image; (**c**) PSR method; (**d**) MIND method; (**e**) ESSD method; (**f**) WLD method; (**g**) NMI method.

Figure 14 shows the TRE for 50 times registration of CT and PD-weighted MR images. From Figure 14, we can see that the proposed method has a lower mean TRE and is more robust than the other methods. In particular, compared with the most competitive MIND method, our method can provide a mean improvement of TRE by 0.33. To compare the performance of the registration methods intuitively, some examples of registration for MR and CT images are shown in Figure 15. Among the five registration methods, the NMI and WLD methods provide the worst registration results. As shown in the red boxes in Figure 14f,g, the registered results are not ideal either for the contour or for the details. The ESSD method performs better than the NMI and WLD methods, but it cannot provide a satisfactory registered result for the weak edges as shown Figure 15e. Besides this, the registered result of the MIND method is worse than that of the PSR method for both contour and image details as shown in the red boxes in Figure 15d. The experiments have demonstrated the superiority of our method in MR and CT image registration.

## 4. Conclusions

In this paper, a novel PCANet-based structural representation method has been proposed for multimodal medical image registration. Compared with the human-designed feature extraction methods, the PCANet can automatically learn the intrinsic features from a large number of medical images through multilevel linear and nonlinear transformations. Distinctively, the proposed method can provide effective structural representations for multimodal images by utilizing the multilevel image features extracted in the various layers of the PCANet. Extensive experiments on Atlas, BrainWeb, and RIRE datasets demonstrate that the proposed method can provide lower TRE values and more satisfactory registered results in terms of human vision than the MIND, ESSD, WLD, and NMI methods. 

Our future work will focus on extending the proposed approach to more challenging data, in particular 3D ultrasound data. The extension of our method is an arduous task due to the serious noise and the unclear anatomical edge in ultrasound images, which pose a challenge for the construction of structural representation. To ensure that the proposed method can be used for the registration of challenging data, the training of the PCANet and the construction of the structural representation will be modified and more clinical samples will be collected to facilitate the training and testing of the PCANet-based registration method.

## Figures and Tables

**Table 1:** TRE for all registration methods performed on the BrainWeb dataset.

Registration Methods	TRE								
	T1-T2			T1-PD			T2-PD		
	Mean	Std	*p*-Value	Mean	Std	*p*-Value	Mean	Std	*p*-Value
/	6.68	2.85	/	6.92	3.06	/	6.21	2.73	/
NMI	1.96	1.74	$2.03\times 10^{-4}$	2.31	1.83	$1.87\times 10^{-5}$	2.43	1.91	$3.03\times 10^{-9}$
WLD	1.84	1.60	$7.06\times 10^{-5}$	2.04	1.79	$4.34\times 10^{-4}$	2.38	1.69	$5.84\times 10^{-10}$
ESSD	1.55	1.24	$4.18\times 10^{-6}$	1.82	1.34	$2.64\times 10^{-6}$	1.67	1.03	$4.02\times 10^{-9}$
MIND	1.22	0.45	$1.02\times 10^{-4}$	1.12	0.52	$1.15\times 10^{-3}$	1.04	0.49	$9.38\times 10^{-3}$
PSR	0.76	0.37	/	0.78	0.46	/	0.73	0.41	/
